# Territory- and Lesion-Specific Endovascular Strategies in Lower Limb Peripheral Artery Disease: A Cohort Study

**DOI:** 10.3390/jcdd13010029

**Published:** 2026-01-04

**Authors:** Thierry Unterseeh, Livio D’Angelo, Mariama Akodad, Youcef Lounes, Hakim Benamer, Benjamin Honton, Antoine Sauguet, Stephane Cook, Mario Togni, Luca Esposito, Gaetano Liccardo, Neila Sayah, Pietro Laforgia, Nicolas Amabile, Thomas Hovasse, Philippe Garot, Antoinette Neylon, Francesca Sanguineti, Stephane Champagne, Ioannis Skalidis

**Affiliations:** 1 Institut Cardiovasculaire Paris-Sud, Hôpital Jacques Cartier, 91300 Massy, France; 2 Department of Cardiology, Clinique Pasteur, 31300 Toulouse, France; 3 Department of Cardiology, HFR—Fribourg Hospital and University, 1752 Fribourg, Switzerland; 4 Department of Advanced Biomedical Sciences, Federico II University of Naples, 80138 Napels, Italy

**Keywords:** peripheral artery disease, endovascular therapy, restenosis, drug-coated balloon, treatment strategy

## Abstract

(1) Background: Endovascular therapy is widely used for lower limb peripheral artery disease (PAD), yet device performance varies across vascular territories due to anatomical and biomechanical differences. This study evaluated territory- and lesion-specific outcomes following contemporary endovascular strategies in a real-world cohort. (2) Methods: This retrospective single-center study included consecutive patients undergoing endovascular revascularization of the iliac, superficial femoral (SFA), or popliteal arteries between 2010 and 2023. The primary endpoint was 12-month binary restenosis (≥50% diameter loss) assessed by duplex ultrasonography, CT angiography, or invasive angiography. Secondary outcomes included target lesion revascularization and procedural complications. Kaplan–Meier analysis was used to evaluate restenosis-free survival. Multivariable Cox models were constructed separately for each vascular territory, adjusting for relevant clinical and anatomical covariates. (3) Results: A total of 283 lesions were included (iliac *n* = 135; SFA *n* = 145; popliteal *n* = 102). At 12 months, restenosis rates differed substantially by treatment modality and arterial territory. In the iliac segment, covered stents demonstrated the lowest restenosis (12.8%), whereas in the SFA, interwoven nitinol stents yielded the most favorable profile (15.4%). In the popliteal artery, drug-coated balloons were associated with the lowest restenosis rate (16.7%). In multivariable analysis, covered stents (iliac), interwoven nitinol stents (SFA), and drug-coated balloons (popliteal) were independently associated with lower restenosis risk. Procedural success was high and complication rates were low. (4) Conclusions: Endovascular device performance is strongly influenced by arterial territory and lesion characteristics. Tailoring the treatment strategy to vessel biomechanics and lesion morphology may optimize mid-term patency in lower limb PAD. Larger prospective studies are warranted to validate these findings.

## 1. Introduction

Peripheral artery disease (PAD) affects more than 200 million individuals worldwide and is associated with substantial functional impairment and increased cardiovascular mortality [1,2]. Endovascular therapy (EVT) has emerged as the first-line treatment for aorto-iliac and femoropopliteal occlusive disease owing to its minimally invasive nature, favorable perioperative profile, and expanding device technology [3,4]. Despite these advantages, long-term durability remains a central limitation of EVT. Restenosis rates frequently exceed 30% at 12 months in complex femoropopliteal and popliteal lesions, particularly in the presence of heavy calcification, long occlusions, or mechanical stress zones [5,6]. These observations underscore the need for more refined, anatomy-specific treatment strategies.

A broad spectrum of endovascular devices is now available—including balloon-expandable and self-expanding covered stents, bare-metal stents (BMSs), interwoven nitinol stents (IWNSs), drug-coated balloons (DCBs), drug-eluting stents (DESs), and plain balloon angioplasty (POBA). Each device class exhibits unique mechanical properties, antiproliferative profiles, and interactions with local vessel compliance and plaque morphology. However, device selection in clinical practice often relies more on operator preference than on structured, lesion- or territory-specific criteria.

Evidence supporting territory-specific performance is already emerging. In the iliac segment, the COBEST randomized trial demonstrated superior patency of covered stents over bare-metal stents in TASC C–D aorto-iliac lesions, highlighting the importance of plaque sealing in heavily diseased, high-flow vessels [7]. Subsequent randomized data from the DISCOVER trial likewise suggested improved outcomes with covered stents in common iliac artery disease, although differences were not statistically significant at two years [8].

In the femoropopliteal region, the biomechanical challenges of torsion, elongation, and external compression have prompted investigation of stent designs optimized for dynamic stress. The STELLA-SUPERA trial reported 24-month primary patency of 77.9% with IWNS and no stent fractures in long TASC C–D lesions, underscoring the mechanical durability of the interwoven architecture [9]. Meta-analyses similarly confirm robust patency of IWNS in real-world practice [10]. Conversely, in the popliteal artery—where mechanical deformation is extreme—DCBs may offer an advantage by avoiding permanent implants and reducing the risk of stent fatigue and fracture.

Despite these insights, contemporary research remains fragmented. Most studies evaluate single device classes within isolated territories, while real-world PAD patients frequently present with multilevel disease and heterogeneous lesion morphologies. Comprehensive assessments integrating lesion characteristics, arterial territory, and treatment modality remain scarce. This gap limits the development of individualized, morphology-guided endovascular strategies.

Importantly, the durability of endovascular interventions is strongly influenced by arterial biomechanics, which vary substantially across vascular territories. The iliac arteries are relatively fixed and exposed to limited deformation, whereas the femoropopliteal and popliteal segments undergo repetitive torsion, elongation, compression, and flexion during daily limb movement. These biomechanical forces interact with lesion length, calcification burden, and device design, directly affecting stent fatigue, fracture risk, and neointimal hyperplasia. Consequently, extrapolating device performance across territories may be inappropriate, reinforcing the need for territory-specific and morphology-guided treatment strategies.

The present cohort study addresses this unmet need by systematically examining the interplay between anatomical territory, lesion morphology, device selection, and 12-month restenosis across the iliac, superficial femoral, and popliteal segments. We hypothesize that aligning endovascular strategy with both arterial biomechanics and lesion complexity results in superior patency outcomes compared with conventional, non-tailored approaches.

## 2. Materials and Methods

### 2.1. Study Design and Population

This retrospective, single-center cohort study included all consecutive patients who underwent endovascular revascularization for symptomatic lower limb peripheral artery disease (PAD) at Claude Galien Hospital, Institut Cardiovasculaire Paris-Sud (Massy/Quincy, France) between January 2010 and December 2023. The study adhered to the Declaration of Helsinki, received approval from the institutional ethics committee, and written informed consent for anonymized data analysis was obtained from all participants.

Patients were eligible if they were at least 18 years of age, presented with symptomatic chronic limb ischemia corresponding to Rutherford categories 2–5, and underwent percutaneous transluminal angioplasty (PTA) with available vascular imaging at approximately 12 months. Individuals were excluded if they underwent concomitant surgical bypass during the index procedure or lacked follow-up imaging suitable for restenosis assessment.

### 2.2. Lesion Classification and Definitions

Arterial lesions were categorized according to three predefined vascular territories: the iliac arteries (common and external iliac segments), the superficial femoral artery (SFA), and the popliteal artery (P1–P3). Lesion morphology was characterized using standard angiographic criteria. Chronic total occlusion (CTO) was defined as complete luminal obstruction with TIMI 0 flow. Severe calcification was identified as circumferential arterial calcification visible on fluoroscopy prior to contrast injection. Dissection was defined as a flow-limiting flap or contrast staining following predilatation or at the final angiographic assessment. In-stent restenosis (ISR) was defined as a ≥50% diameter reduction within a previously implanted stent.

In patients with multilevel PAD, one index lesion per anatomical territory was selected for analysis, prioritizing the segment deemed most clinically relevant based on its hemodynamic significance, proximal location, and relationship to presenting symptoms. Each lesion was analyzed independently, irrespective of whether the intervention was unilateral or bilateral.

### 2.3. Procedural Techniques and Device Categories

All procedures were performed under fluoroscopic guidance by experienced interventionalists using either femoral or radial access. Device selection was left to operator discretion and included covered stents, interwoven nitinol stents (IWNSs), drug-eluting stents (DESs), conventional bare-metal stents (BMSs), drug-coated balloons (DCBs) with or without bailout stenting, and plain balloon angioplasty (POBA) without drug coating or stent implantation. Predilatation, postdilatation, vessel preparation techniques, and any bailout stenting were systematically recorded. Technical success was defined as residual stenosis < 30% without flow-limiting dissection or major periprocedural complications.

Vessel preparation strategies, including predilatation and postdilatation, were performed at the operator’s discretion based on lesion length, calcification severity, and angiographic appearance. Bailout stenting following balloon angioplasty or drug-coated balloon treatment was reserved for flow-limiting dissection or significant residual stenosis. Periprocedural antithrombotic management followed institutional protocols and contemporary guideline recommendations, with all patients receiving antiplatelet therapy unless contraindicated.

### 2.4. Outcomes and Follow-Up

The primary endpoint was binary restenosis at 12 months, defined as a ≥50% diameter stenosis on follow-up duplex ultrasonography, computed tomography angiography, or invasive angiography, according to institutional surveillance protocols. Follow-up imaging was performed according to routine clinical practice and was therefore not standardized to a single modality. Duplex ultrasonography represented the predominant follow-up method, while computed tomography angiography or invasive angiography was used when clinically indicated. Duplex ultrasound assessments were performed according to standardized institutional criteria, including established peak systolic velocity ratio thresholds for the definition of ≥50% restenosis.

Secondary endpoints included target lesion revascularization (TLR), procedural success, and periprocedural complications such as access-site hematoma, pseudoaneurysm, dissection, distal embolization, and acute thrombosis. All imaging studies were reviewed by two independent investigators blinded to the treatment modality.

### 2.5. Statistical Analysis

Baseline, anatomical, and procedural characteristics were summarized using descriptive statistics. Continuous variables were presented as mean ± standard deviation or median (interquartile range) and compared across groups using one-way ANOVA or the Kruskal–Wallis test, as appropriate. Categorical variables were expressed as frequencies and percentages and compared using the chi-square or Fisher’s exact test. Because bailout stenting occurred infrequently, DCB angioplasty performed with or without additional BMS implantation was analyzed as a single therapeutic category.

Restenosis-free survival was assessed using Kaplan–Meier methodology with comparisons between treatment strategies performed using the log-rank test. To identify independent predictors of restenosis within each vascular territory, territory-specific multivariable Cox proportional hazards models were constructed. Covariates were selected a priori based on established clinical relevance and included age, sex, diabetes mellitus, lesion length, reference vessel diameter, severe calcification, dissection, ISR, and treatment modality. All analyses were two-sided, and a *p*-value < 0.05 was considered statistically significant. Statistical analyses were conducted using R version 4.3.2 (R Foundation for Statistical Computing, Vienna, Austria).

### 2.6. Ethical Considerations

The study protocol was approved by the local institutional ethics committee, and written informed consent was obtained from all participants. Data collection and analysis complied with institutional data protection policies and applicable regulatory standards.

## 3. Results

### 3.1. Study Population and Lesion Characteristics

A total of 283 patients undergoing endovascular treatment for symptomatic lower limb peripheral artery disease were included. Baseline clinical and anatomical characteristics are summarized in Table 1. Lesions were distributed across the iliac (47.7%), superficial femoral (51.2%), and popliteal segments (36.1%). Chronic total occlusion (CTO) was present in 33.6% of lesions, while severe calcification was identified in 62.5%. In-stent restenosis (ISR) accounted for 5.3% of treated lesions. Median lesion length was 64 mm (IQR 30–80 mm), and the mean reference vessel diameter was 7.0 ± 1.6 mm.

Overall, the cohort represented a population with advanced atherosclerotic disease burden. More than one-third of lesions were chronic total occlusions, and nearly two-thirds exhibited severe calcification, underscoring the anatomical complexity treated in routine clinical practice. Prior lower limb revascularization was frequent across all territories, indicating a substantial proportion of patients with recurrent or progressive disease.

Baseline differences across arterial territories followed expected anatomical patterns. Iliac lesions were characterized by larger reference vessel diameters and shorter lesion lengths, whereas superficial femoral and popliteal lesions were longer, more frequently chronic total occlusions, and more commonly associated with severe calcification. Territory-specific baseline differences in lesion length, vessel diameter, CTO prevalence, and calcification severity are detailed in Table 1.

**Table 1:** Baseline clinical and lesion characteristics stratified by arterial territory.

Variable	Iliac (*n* = 135)	SFA (*n* = 145)	Popliteal (*n* = 102)
Age, years (mean ± SD)	69.2 ± 10.4	66.8 ± 11.6	65.3 ± 11.9
Male sex, *n* (%)	110 (81.5%)	115 (79.3%)	82 (80.4%)
Diabetes mellitus, *n* (%)	45 (33.3%)	60 (41.4%)	42 (41.2%)
Current/former smoker, *n* (%)	100 (74.1%)	105 (72.4%)	78 (76.5%)
Chronic kidney disease, *n* (%)	28 (20.7%)	32 (22.1%)	24 (23.5%)
Hypertension, *n* (%)	112 (83.0%)	124 (85.5%)	88 (86.3%)
Hyperlipidemia, *n* (%)	90 (66.7%)	101 (69.7%)	70 (68.6%)
Coronary artery disease, *n* (%)	62 (45.9%)	71 (49.0%)	48 (47.1%)
History of stroke/TIA, *n* (%)	16 (11.9%)	19 (13.1%)	13 (12.7%)
Prior lower limb revascularization, *n* (%)	36 (26.7%)	44 (30.3%)	29 (28.4%)
Chronic total occlusion (CTO), *n* (%)	30 (22.2%)	62 (42.8%)	45 (44.1%)
Severe calcification, *n* (%)	85 (63.0%)	90 (62.1%)	64 (62.7%)
In-stent restenosis (ISR), *n* (%)	10 (7.4%)	5 (3.4%)	3 (2.9%)
Post-predilatation or final dissection, *n* (%)	12 (8.9%)	25 (17.2%)	18 (17.6%)
Median lesion length, mm [IQR]	55 [28–72]	70 [40–90]	60 [35–78]
Reference vessel diameter, mm (mean ± SD)	8.2 ± 1.1	6.4 ± 1.3	5.9 ± 1.2

Data are presented as mean ± standard deviation or median [interquartile range] for continuous variables, and number (percentage) for categorical variables. BTK lesions were excluded from this table due to insufficient representation. SFA, superficial femoral artery; CTO, chronic total occlusion; ISR, in-stent restenosis.

### 3.2. Treatment Strategies Across Arterial Territories

Device use varied significantly by arterial segment (Table 2). In the iliac arteries, covered stents and bare-metal stents (BMSs) were the predominant strategies, whereas interwoven nitinol stents (IWNSs) and drug-coated balloons (DCBs) were preferred in the superficial femoral and popliteal arteries, respectively. Drug-eluting stents (DESs) were selectively used across territories but represented a minority of overall treatments.

### 3.3. Primary Outcome: 12-Month Restenosis

**Figure 1:**
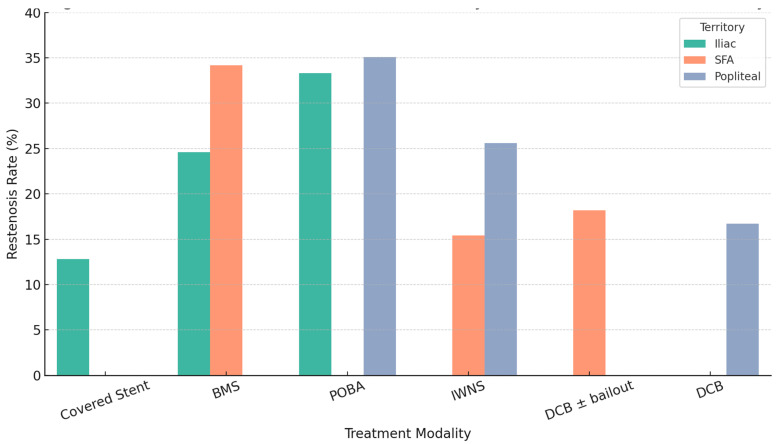
Observed 12-Month Restenosis Rates by Treatment Modality and Arterial Territory. Bar plot showing unadjusted binary restenosis rates at 12 months across three vascular territories (iliac, SFA, and popliteal artery), stratified by endovascular treatment modality. Covered stents demonstrated the lowest restenosis rate in the iliac artery (12.8%), IWNSs in the SFA (15.4%), and DCBs in the popliteal artery (16.7%). POBA and conventional BMSs were associated with higher restenosis rates across all territories. These findings support the use of lesion- and territory-specific treatment strategies in lower limb peripheral interventions. BMS, bare-metal stent; DCB, drug-coated balloon; IWNS, interwoven nitinol stent; POBA, plain old balloon angioplasty; SFA, superficial femoral artery.

At 12-month imaging follow-up, the overall restenosis rate was 28.7%. Restenosis varied substantially by both arterial territory and treatment modality (Table 3) (Figure 1).

Iliac Artery: Covered stents demonstrated the lowest restenosis rate (12.8%), followed by BMS (24.6%) and POBA (33.3%). No restenosis events occurred among iliac lesions treated with DES.

Superficial Femoral Artery: IWNS achieved the most favorable patency (15.4%), followed by DCB ± bailout stenting (18.2%). Conventional BMS was associated with significantly higher restenosis (34.2%). As in the iliac territory, no restenosis was observed with DES.

Popliteal Artery: DCB angioplasty yielded the lowest restenosis rate (16.7%), whereas POBA and IWNS were associated with higher restenosis (35.1% and 25.6%, respectively). DES use in this segment resulted in no observed restenosis events, although numbers were limited.

Across territories, patterns of restenosis aligned closely with the mechanical and biological characteristics of each treatment modality, as well as with the known biomechanical stresses of each arterial segment.

### 3.4. Adjusted Predictors of Restenosis

Multivariable Cox proportional hazards models, constructed separately for each vascular territory, identified the optimal endovascular strategy for each segment (Table 4):

Iliac artery: covered stents were independently associated with lower restenosis risk compared with BMS (HR 0.51, 95% CI 0.29–0.91; *p* = 0.02).

Superficial femoral artery: IWNS significantly reduced restenosis risk compared with BMS (HR 0.56, 95% CI 0.31–0.98; *p* = 0.04).

Popliteal artery: DCB angioplasty was independently protective relative to POBA (HR 0.48, 95% CI 0.26–0.89; *p* = 0.02).

DES were not included in Cox models because zero restenosis events prevented hazard estimation; however, outcomes in this subgroup were consistently favorable.

**Figure 2:**
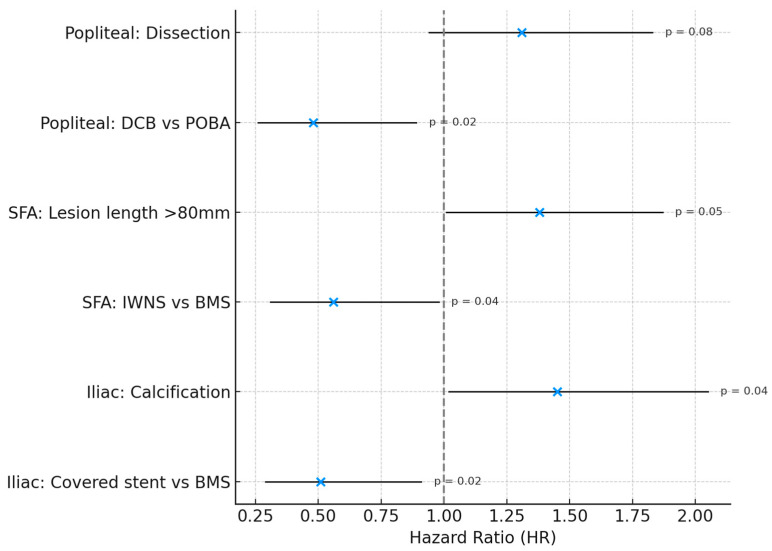
Multivariable Predictors of Restenosis at 12 Months: Forest Plot. Forest plot illustrating hazard ratios (HRs) and 95% confidence intervals (CIs) for independent predictors of 12-month restenosis across three vascular territories (iliac, superficial femoral artery [SFA], and popliteal). Hazard ratios were derived from Cox proportional hazards regression models adjusted for age, sex, diabetes, lesion length, vessel diameter, severe calcification, dissection, and in-stent restenosis. A vertical reference line at HR = 1.0 indicates no association with restenosis risk. Predictors with *p* < 0.05 were considered statistically significant. BMS, bare-metal stent; DCB, drug-coated balloon; IWNS, interwoven nitinol stent; POBA, plain old balloon angioplasty; SFA, superficial femoral artery.

These adjusted analyses reinforce that device-territory alignment—covered stents in the iliac arteries, IWNSs in the SFA, and DCBs angioplasty in the popliteal artery—yields the most durable 12-month patency (Figure 2).

### 3.5. Restenosis-Free Survival

Kaplan–Meier analysis showed clear separation of restenosis-free survival curves across treatment modalities within each vascular segment. Covered stents in the iliac arteries, IWNSs in the SFA, and DCBs angioplasty in the popliteal segment consistently demonstrated superior survival free from restenosis. These time-to-event patterns were fully concordant with the binary 12-month restenosis results and multivariable hazard ratios.

### 3.6. Secondary Outcomes and Procedural Safety

Technical success was achieved in 92.6% of procedures. Target lesion revascularization at 12 months did not significantly differ across territories or treatment strategies. Bailout stenting was required in 6.7% of lesions initially treated with POBA or DCB, primarily due to flow-limiting dissections.

The overall periprocedural complication rate was 6.3%, with low rates of access-site hematoma (2.5%), pseudoaneurysm (1.4%), and acute thrombosis (0.7%). Flow-limiting dissection requiring unplanned stent placement occurred in 2.4% of cases. No meaningful differences were observed in complication rates across treatment modalities.

## 4. Discussion

In this single-center, retrospective cohort reflecting the real-world experience of a medium-volume center, we explored territory- and lesion-specific patterns of endovascular treatment for lower limb peripheral artery disease. Given the modest sample size within individual device categories, this study was not designed to provide definitive comparative evidence, but rather to describe consistent, hypothesis-generating patterns of device performance across vascular territories over 12-month follow-up.

The lower restenosis rates observed with covered stents in the iliac arteries are concordant with prior reports showing that plaque exclusion and improved radial strength offer advantages in this relatively stable, large-caliber vessel. In the superficial femoral artery, interwoven nitinol stents (IWNS) demonstrated favorable 12-month patency compared with conventional bare-metal stents. This finding is compatible with the known flexibility and resistance to fracture of IWNS in a segment subjected to substantial elongation, torsion, and compression during limb movement.

In the popliteal artery, drug-coated balloon angioplasty resulted in lower restenosis rates relative to POBA and IWNS. The absence of a permanent implant may be beneficial in this highly mobile vascular segment, where stent fracture and mechanical fatigue remain concerns. Although the number of DES-treated lesions was small, no restenosis was observed in this subgroup; however, this selective use prevents broader conclusions. The absence of restenosis among DES-treated lesions must be interpreted cautiously, as DESs were selectively implanted in anatomically favorable cases. These findings likely reflect selection bias rather than a definitive advantage of DES.

Importantly, the overall patterns observed in this study are not intended to redefine practice but rather to highlight that device performance is context-dependent and may vary according to arterial territory and lesion morphology. The results support the general principle that individualized strategy selection can influence mid-term outcomes, even within the constraints of routine-care decision making. The procedural complication rate was low and comparable to published literature, reflecting the safety of contemporary endovascular approaches across device categories.

Overall, while the findings are not definitive, they provide additional real-world insight into how endovascular devices perform across different arterial territories and may help inform daily decision making in similar patient populations. The observed associations should not be interpreted as causal or as evidence of device superiority, but rather as reflections of contemporary real-world decision-making.

### Limitations

This study has several important limitations. First, it is a retrospective analysis from a single medium-volume center, which limits external validity. The findings reflect local practice patterns, operator expertise, and device availability, and therefore may not be generalizable to other institutions. The relatively small number of lesions within individual device categories reflects the experience of a single, medium-volume center and limits the ability to draw definitive comparative conclusions. Accordingly, the present findings should be considered descriptive and hypothesis-generating rather than practice-defining. Moreover, device selection was not randomized and was driven by operator judgment and lesion characteristics, resulting in unavoidable selection bias and confounding by indication that cannot be fully corrected despite multivariable adjustment. Second, device selection was not randomized and was influenced by operator judgment, lesion complexity, and anatomical considerations. This introduces confounding by indication, which cannot be fully accounted for even with adjusted analyses.

Third, the sample size—particularly within individual device subgroups—was relatively small. Some comparisons, including those involving DESs, should be interpreted with caution due to limited statistical power. Fourth, although all lesions included had 12-month imaging follow-up, the imaging modality was not standardized and varied between duplex ultrasonography, computed tomography angiography, and invasive angiography. Differences in sensitivity across modalities may influence restenosis detection. Fifth, certain potentially relevant anatomical variables—such as plaque morphology, eccentricity, or precise lesion biomechanics—were not systematically recorded and therefore not incorporated into the analysis. Although all lesions had 12-month imaging follow-up, the imaging modality was not standardized and varied between duplex ultrasonography, computed tomography angiography, and invasive angiography. Differences in sensitivity and specificity across modalities may have influenced restenosis detection and represent an additional source of variability Finally, the observational design precludes any causal inference about the superiority of one device over another. The results should be considered hypothesis-generating rather than definitive.

## 5. Conclusions

In this single-center, real-world cohort, endovascular device performance differed across arterial territories. Covered stents in the iliac arteries, interwoven nitinol stents in the SFA, and drug-coated balloons in the popliteal segment showed the most favorable 12-month patency within their respective regions. While these observations require cautious interpretation due to the study’s retrospective design and limited sample size, they support the importance of aligning treatment strategy with lesion anatomy and vascular biomechanics. These findings reflect the real-world experience of a medium-volume center and should be interpreted as hypothesis-generating rather than definitive guidance for device selection. Larger prospective studies are needed to confirm these findings and guide optimal device selection in lower limb PAD.

## Figures and Tables

**Table 2:** Distribution of treatment modalities by arterial territory.

Treatment Modality	Iliac (*n* = 135)	SFA (*n* = 145)	Popliteal (*n* = 102)
Covered stents	47 (34.8%)	0 (0.0%)	0 (0.0%)
Bare-metal stents (BMSs)	42 (31.1%)	28 (19.7%)	5 (4.9%)
Drug-eluting stents (DESs)	12 (8.9%)	46 (31.7%)	8 (7.8%)
Interwoven nitinol stents (IWNSs)	0 (0.0%)	59 (40.9%)	15 (14.4%)
Drug-coated balloon (DCB) ± bailout	8 (5.9%)	40 (27.3%)	47 (45.6%)
Plain old balloon angioplasty (POBA)	28 (20.7%)	18 (12.4%)	28 (27.2%)
No device implanted (e.g., balloon only, no complications)	10 (7.4%)	12 (8.3%)	7 (6.9%)

Values represent the number of lesions treated and corresponding percentages. IWNSs, interwoven nitinol stents; DCB, drug-coated balloon; DES, drug-eluting stent, POB, plain old balloon angioplasty; SFA, superficial femoral artery.

**Table 3:** Twelve-month binary restenosis rates stratified by arterial territory and treatment modality.

Arterial Territory	Treatment Modality	12-Month Restenosis Rate (%)	95% CI
Iliac artery	Covered stent	12.8%	8.2–19.4%
	Bare-metal stent (BMS)	24.6%	17.5–33.3%
	Drug-eluting stent (DES)	0.0%	0.0–7.4% *
	Plain old balloon angioplasty (POBA)	33.3%	19.8–50.1%
SFA	Interwoven nitinol stent (IWNS)	15.4%	9.3–24.3%
	Drug-eluting stent (DES)	0.0%	0.0–5.2% *
	Drug-coated balloon (DCB) ± bailout stenting	18.2%	11.0–28.5%
	Conventional BMS	34.2%	23.5–46.8%
Popliteal artery	DCB angioplasty	16.7%	10.1–26.3%
	Drug-eluting stent (DES)	0.0%	0.0–12.3% *
	IWNS	25.6%	15.2–40.0%
	POBA	35.1%	23.3–49.3%

This table presents raw restenosis percentages by treatment strategy within each anatomical zone. Comparative statistical analyses are reported separately in the main text and in Table 4. * Restenosis rates for drug-eluting stents were 0.0% across all territories. However, due to limited sample size and potential selection bias, DES were excluded from multivariable modeling. SFA, superficial femoral artery; POBA, plain old balloon angioplasty; DCB, drug-coated balloon; IWNS, interwoven nitinol stent; BMS, bare-metal stent.

**Table 4:** Multivariable Cox regression analysis evaluating the association between treatment modality and 12-month binary restenosis, stratified by arterial territory.

Arterial Territory	Treatment Comparison	Hazard Ratio (HR)	95% Confidence Interval	*p*-Value
Iliac artery	Covered stent vs. BMS	0.51	0.29–0.91	0.02
SFA	IWNS vs. conventional BMS	0.56	0.31–0.98	0.04
Popliteal artery	DCB vs. POBA	0.48	0.26–0.89	0.02

Each model was adjusted for age, sex, diabetes mellitus, lesion length, reference vessel diameter, severe calcification, post-procedural dissection, and in-stent restenosis (ISR). Hazard ratios (HRs) are presented with 95% confidence intervals (CIs). Reference groups: BMS in iliac and SFA territories; POBA in the popliteal segment. BMS, bare-metal stent; IWNS, interwoven nitinol stent; DCB, drug-coated balloon; POBA, plain old balloon angioplasty; SFA, superficial femoral artery.

## Data Availability

Data available upon request to the corresponding author.

## References

[1] Fowkes F.G.R., Rudan D., Rudan I., Aboyans V., Denenberg J.O., McDermott M.M., Norman P.E., Sampson U.K.A., Williams L.J., Mensah G.A. (2013). Comparison of global estimates of prevalence and risk factors for peripheral artery disease in 2000 and 2010: A systematic review and analysis. Lancet.

[2] Criqui M.H., Aboyans V. (2015). Epidemiology of peripheral artery disease. Circ. Res..

[3] Norgren L., Hiatt W.R., Dormandy J.A., Nehler M.R., Harris K.A., Fowkes F.G., Bell K., Caporusso J., Du-rand-Zaleski I., TASC II Working Group (2007). Inter-Society Consensus for the Management of Peripheral Arterial Disease (TASC II). Eur. J. Vasc. Endovasc. Surg..

[4] Schillinger M., Sabeti S., Loewe C., Dick P., Amighi J., Mlekusch W., Schlager O., Cejna M., Lammer J., Minar E. (2006). Balloon angioplasty versus implantation of nitinol stents in the superficial femoral artery. N. Engl. J. Med..

[5] Davaine J.M., Azéma L., Guyomarch B., Chaillou P., Costargent A., Patra P., Lambert G., Gouëffic Y. (2012). One-year clinical outcome after primary stenting for Trans-Atlantic Inter-Society Consensus (TASC) C and D femoropopliteal lesions (the STELLA “STEnting Long de L’Artère fémorale superficielle” cohort). Eur. J. Vasc. Endovasc. Surg..

[6] Van Meirvenne E., Reyntjens P., Tielemans Y. (2022). Self-expanding interwoven nitinol stent in severe femoropopliteal arterial disease. Real life results of the Supera Peripheral Stent System^®^. Acta Chir. Belg..

[7] Mwipatayi B.P., Thomas S., Wong J., Temple S.E., Vijayan V., Jackson M., Burrows S.A., Covered Versus Balloon Expandable Stent Trial (COBEST) Co-investigators (2011). A comparison of covered vs bare expandable stents for the treatment of aortoiliac occlusive disease. J. Vasc. Surg..

[8] Bekken J.A., Vroegindeweij D., Vos J.A., de Vries J.P.M., Lardenoije J.W.H.P., Petri B.J., Pierie M.E.N., van Weel V., Teijink J.A.W., Fioole B. (2023). Editor’s Choice—Two Year Results of the Randomised DISCOVER Trial Comparing Covered Versus Bare Metal Stents in the Common Iliac Artery. Eur. J. Vasc. Endovasc. Surg..

[9] Nasr B., Gouailler F., Marret O., Guillou M., Chaillou P., Guyomarc’h B., Maurel B., Gouëffic Y. (2022). Treatment of Long Femoropopliteal Lesions with Self-Expanding Interwoven Nitinol Stent: 24 Month Outcomes of the STELLA-SUPERA Trial. J. Endovasc. Ther..

[10] Bontinis V., Antonopoulos C.N., Bontinis A., Koutsoumpelis A., Giannopoulos A., Ktenidis K. (2022). A systematic review and meta-analysis of Supera interwoven nitinol stents for the treatment of infrainguinal peripheral arterial disease. J. Cardiovasc. Surg..

